# Advances in Doxorubicin Chemotherapy: Emerging Polymeric Nanocarriers for Drug Loading and Delivery

**DOI:** 10.3390/cancers17142303

**Published:** 2025-07-10

**Authors:** Abhi Bhadran, Himanshu Polara, Godwin K. Babanyinah, Sruthy Baburaj, Mihaela C. Stefan

**Affiliations:** 1 Chemistry and Biochemistry, The University of Texas at Dallas, Richardson, TX 75080, USA; himanshu.polara@utdallas.edu (H.P.); godwin.babanyinah@utdallas.edu (G.K.B.); mihaela@utdallas.edu (M.C.S.); 2 Center for Photochemical Sciences, Bowling Green State University, Bowling Green, OH 43403, USA; sruthyb@bgsu.edu

**Keywords:** cancer, chemotherapy, doxorubicin, cardiotoxicity, polymeric nanocarriers, polymeric micelles, drug delivery, loading capacity

## Abstract

Background/Objectives: Effective and targeted delivery of doxorubicin (DOX) remains a significant challenge due to its dose-limiting cardiotoxicity and systemic side effects. Liposomal formulations like Doxil^®^ have improved tumor targeting and reduced toxicity, but issues such as limited stability, poor release control, and insufficient site-specific delivery persist. As a result, there is a growing interest in advanced drug delivery systems, particularly polymeric nanocarriers, which offer biocompatibility, tunable properties, and ease of fabrication. Methods: This review is organized into two key sections. The first section provides a comprehensive overview of DOX, including its mechanism of action, clinical challenges, and the limitations of current chemotherapy approaches. The second section highlights recent advances in polymeric nanocarriers for DOX delivery, focusing on polymeric micelles as well as other promising systems like hydrogels, dendrimers, polymersomes, and polymer–drug conjugates. Results: Initial discussions explore current strategies enhancing DOX’s clinical translation, including methods to address cardiotoxicity and multidrug resistance. The latter part presents recent studies that report improved drug loading efficiency in polymeric nanocarriers through techniques such as core/shell modifications, enhanced hydrophobic interactions, and polymer–drug conjugation. Conclusions: Despite notable progress in polymeric nanocarrier-based DOX delivery, challenges like limited circulation time, immunogenicity, and manufacturing scalability continue to hinder clinical application. Continued innovation in this field is crucial for the development of safe, effective, and clinically translatable polymeric nanocarriers for cancer therapy.

## Simple Summary

Doxorubicin is a potent chemotherapeutic agent commonly used in the treatment of various cancers. However, its clinical application is limited by poor aqueous solubility and dose-dependent cardiotoxicity. Polymeric nanocarriers have emerged as promising delivery systems to address these issues by improving drug stability, release control, and bioavailability. This review summarizes recent advances in polymeric nanocarrier design to enhance doxorubicin delivery and efficacy while minimizing associated limitations.

## 1. Introduction

Cancer continues to be a major health concern in the United States, with projections from the American Cancer Society indicating that it will remain the second leading cause of death in 2025. An estimated 2,041,910 new cases and 618,120 cancer-related deaths are expected during this year [1]. As cancer incidence rates rise, the demand for effective therapeutic strategies becomes increasingly critical. Chemotherapy remains one of the most widely used and effective approaches for treating various types of cancer, aimed at inhibiting the growth, proliferation, and metastasis of malignant cells [2,3]. Since 1949, the U.S. Food and Drug Administration (FDA) has approved hundreds of anticancer drugs, greatly improving treatment options and advancing cancer therapy.

Among the many chemotherapeutic agents, doxorubicin (DOX) is a widely utilized and effective drug for treating a broad spectrum of cancers, including breast, ovarian, bladder, and lung [4]. DOX belongs to the anthracycline family, a class of drugs known for their potent anticancer activity. Its mechanism of action involves inhibiting topoisomerase II, an enzyme responsible for managing DNA supercoiling during replication [5]. In addition, DOX can intercalate into DNA, further disrupting cellular processes and interfering with transcription and replication. This dual mechanism enhances its cytotoxic efficacy [6]. DOX is particularly valuable in the treatment of early-stage and metastatic breast cancer, where it offers a survival advantage over non-anthracycline adjuvant regimens [7]. Its broad applicability and proven efficacy have made it a key component of chemotherapy regimens.

Despite its effectiveness, the clinical use of DOX is significantly hindered by its poor water solubility and severe toxicity to healthy tissues. DOX’s limited aqueous solubility makes its formulation and delivery challenging, often requiring solubilizing agents or carriers, which can introduce additional side effects [2,3,8]. A significant concern with DOX therapy is its systemic toxicity, particularly its cardiotoxic effects, which can lead to acute and chronic heart complications, including cardiomyopathy and heart failure [6]. Clinical data show that approximately 7% of patients develop cardiomyopathy at cumulative doses exceeding 550 mg/m^2^ [9,10]. Moreover, long-term administration of DOX is associated with neurotoxicity and nephrotoxicity, contributing to chronic brain and kidney damage [11,12,13]. These adverse effects significantly limit the dosage and duration of DOX treatment, reducing its therapeutic potential.

To overcome the limitations of DOX, extensive research has been dedicated to developing advanced drug delivery systems aimed at improving its solubility and reducing off-target toxicity. These systems can bridge the barrier between the drug and tumor sites by staying in the blood circulatory system for a prolonged time and preventing the drug from being washed out. Various drug delivery systems, such as liposomal encapsulation, polymeric nanoparticles, micelles, and prodrug formulations, have been investigated to optimize the pharmacokinetics and biodistribution of DOX. These approaches aim to enhance solubility and improve tumor targeting while reducing adverse effects on healthy tissues. A notable example is Doxil^®^, the first FDA-approved nanodrug, which utilizes a liposomal formulation to improve therapeutic efficacy and lower cardiotoxicity by promoting drug accumulation in tumors through the enhanced permeability and retention (EPR) effect [14,15]. Doxil^®^ encapsulates doxorubicin (DOX) using a remote (active) loading method that exploits pH and ion gradients across the liposomal membrane. Typically, liposomes are preloaded with an ammonium sulfate solution, creating a transmembrane pH gradient. When DOX is added externally, it diffuses into the liposome in its neutral form and becomes protonated inside the acidic core, leading to its ion trapping and precipitation as sulfate salts. This mechanism enables high drug-loading efficiency (>90%) and stable encapsulation, which contributes to Doxil’s extended circulation time and reduced cardiotoxicity [14]. Likewise, polymer-based drug delivery systems have shown promise in increasing DOX solubility and enabling controlled release, thereby minimizing systemic toxicity [16,17].

Polymeric nanocarriers are effective and promising drug delivery tools that control the function and biodistribution of encapsulated drug molecules [18,19]. Polymeric nanocarriers demonstrate unique advantages. These include convenient surface modification by chemical transformation, compatibility in delivering a wide range of bioactive agents, and the ability to form various nanostructures such as polymeric micelles, dendrimers, nanogels, and nanocapsules [18,20,21,22,23]. Among them, several polymeric micellar formulations are undergoing clinical trials for neoplastic and cancer treatment, including NK012, NK105, NC-6004, NC-4016, and NK911 [24,25,26]. An antiviral formulation, Vivagel, containing dendrimer SPL7013, and a dendrimer-based conjugate formulation, DEPTM-Docetaxel, are also undergoing clinical trials [24,27,28]. However, the clinical translation of polymeric dendrimer nanocarriers is not promising due to heterogeneity in formulation, premature leakage, and irreproducible pharmacokinetics [29,30]. Conversely, the performance of linear and branched polymers has significantly improved over the years.

Polymeric nanocarriers can be directed to tumor sites via passive or active targeting strategies. Passive targeting leverages the EPR effect, which allows nanosized (10–200 nm) particles to accumulate preferentially in tumor tissues due to their leaky vasculature and impaired lymphatic drainage [15]. However, the extent of the EPR effect varies significantly across tumor types, disease stages, and individual patients, limiting its consistency and clinical reliability [31]. To address these limitations, polymeric nanocarriers, though still fundamentally dependent on the EPR effect for tumor accumulation, incorporate additional design strategies such as active targeting and stimuli-responsive release mechanisms to enhance their performance. Various targeting moieties have been employed to enhance the specificity of DOX-loaded polymeric nanocarriers toward cancer cells. Small molecule ligands such as folic acid, hyaluronic acid, and galactose target overexpressed receptors like folate receptor, CD44, and ASGPR, respectively [32]. Peptide ligands, including RGD, GE11, and TAT peptides, offer receptor-specific binding and improved cellular penetration [33]. Additionally, monoclonal antibodies and fragments, such as trastuzumab (anti-HER2), cetuximab (anti-EGFR), and anti-CD44, provide high specificity for tumor-associated antigens [34]. Aptamers, like AS1411 targeting nucleolin, also serve as antibody alternatives with comparable affinity and lower immunogenicity [35]. These strategies collectively improve tumor accumulation, reduce systemic toxicity, and enhance the therapeutic efficacy of DOX-based nanocarrier systems.

Despite these promising developments, polymeric nanocarriers, particularly polymeric micelles, still face critical challenges that limit their widespread clinical translation. Among these, the most notable drawback is their typically low drug loading capacity (DLC), which restricts therapeutic efficacy and necessitates higher dosages that may cause off-target toxicity. The inherently small hydrophobic core of micelles limits the amount of drug that can be efficiently encapsulated. Furthermore, weak drug–polymer interactions and premature drug release further compromise the effectiveness of these systems. Significant efforts have been directed toward optimizing micelle composition and architecture to address these limitations. Conventional micelles, such as PEG–PLA or PEG–PCL systems, typically exhibit low DLC (~5–10%) and lack controlled release mechanisms. In contrast, stimuli-responsive micelles are capable of releasing their payload in response to pH or redox gradients that offer improved drug loading (~15–30%) and superior therapeutic selectivity [36,37,38]. For example, the diblock polymers poly(ethylene glycol) methyl ether-b-poly(N,N-diethylaminoethyl methacrylate) (MPEG–PDEAEMA) and poly(ethylene glycol) methyl ether-b-polycaprolactone (MPEG–PCL) were co-micellized to simplify the synthetic process and to improve DLC and pH-responsive drug release behavior [39]. The resulting DLC value of the polymer was in the range of 20–30% which was achieved by simply mixing the above two polymers in different ratios. Other strategies such as polymer functionalization, introduction of aromatic pendant groups, and co-loading of synergistic agents like polyphenols have led to substantial improvements in DLC. In this context, we aim to summarize some of the most recent advancements in polymeric nanocarriers, focusing on polymeric micelles where enhanced DLC was successfully achieved, offering new directions for more effective drug delivery systems.

## 2. Doxorubicin

**Figure 1:**
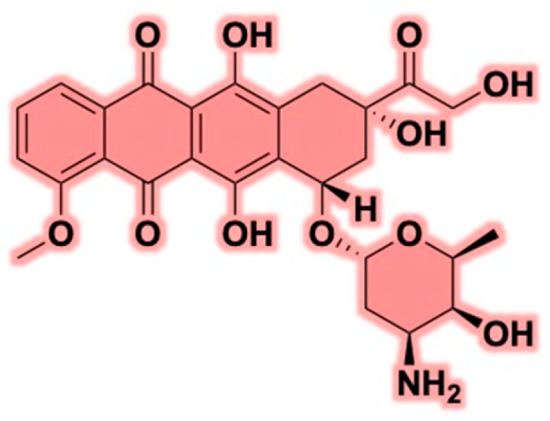
Structure of doxorubicin.

DOX is a potent anthracycline chemotherapeutic agent extensively used in oncology to treat various solid and hematological malignancies (Figure 1). Its efficacy has been well-documented against cancers such as breast, ovarian, lung, and bladder cancers, as well as multiple myeloma and various leukemia types [40,41]. The mechanisms through which DOX exerts its cytotoxic effects are multifaceted, involving pathways that inhibit cancer cell proliferation and survival. Primary mechanisms include DNA intercalation, topoisomerase II inhibition, and reactive oxygen species (ROS) production [42]. These mechanisms function in a coordinated manner, where DNA intercalation and topoisomerase II inhibition lead to double-strand DNA breaks [43]. ROS generation exacerbates genomic instability, amplifying the cytotoxic effects of DOX. However, despite its significant anticancer activity, the clinical application of DOX is hindered by severe off-target toxicities, particularly cardiotoxicity, alongside the development of multidrug resistance (MDR) [44].

DOX belongs to the anthracycline class of antibiotics, initially derived from *Streptomyces peucetius* in the 1960s [45]. Its precursor, daunorubicin, demonstrated potent anticancer activity but was associated with severe cardiotoxicity, leading to the development of DOX as a safer alternative [46]. Structurally, DOX consists of a polyketide-derived tetracenequinone core linked to an amino sugar, daunosamine, contributing to its interactions with hydrophilic and hydrophobic environments. The anthraquinone chromophore plays a crucial role in redox cycling and ROS production, influencing anticancer efficacy and toxicity [47]. DOX is moderately lipophilic (Log P ~1.3) and cationic at physiological pH, allowing interaction with negatively charged cellular components such as membranes and nucleic acids [48,49]. It exhibits moderate water solubility (~1–25 mg/mL depending on pH and formulation), and due to its low permeability (6.72 × 10^−7^ cm/s), with an efflux ratio of 6.6, and limited oral absorption, it is administered intravenously [50]. According to the Biopharmaceutics Classification System (BCS), DOX is generally categorized as a Class III, indicating low permeability and moderate to low solubility, posing challenges for oral delivery [51]. These physicochemical properties critically influence its formulation strategies, directly impacting its effectiveness, stability, and suitability for clinical translation. Current research aims to refine DOX formulations, develop cardioprotective measures, and explore combination therapies to optimize its benefits. Integrating nanotechnology, targeted drug delivery, and personalized medicine offers promising avenues for improving DOX-based chemotherapy, ensuring better patient outcomes while minimizing adverse effects [52,53,54,55].

### 2.1. Mechanisms of Action

**Figure 2:**
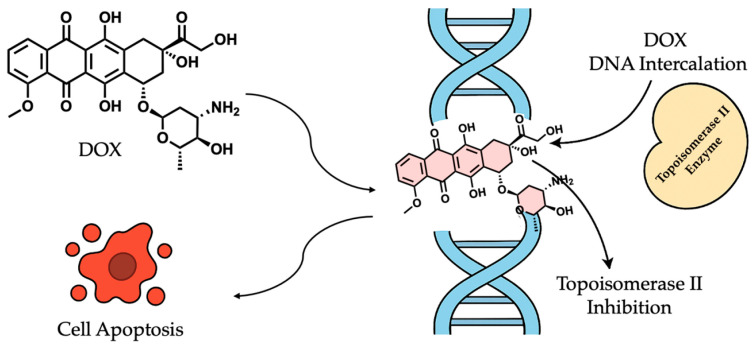
Key mechanism of action of doxorubicin leading to cell apoptosis.

One key mechanism by which DOX induces cytotoxicity is through DNA intercalation. The drug intercalates between base pairs in the DNA helix, disrupting its structure and impairing crucial cellular processes such as transcription and replication. This disruption leads to genomic instability, activation of cell cycle checkpoints, and, ultimately, apoptosis [56,57]. Additionally, DOX inhibits topoisomerase II, a critical enzyme that alleviates DNA supercoiling during replication. By stabilizing the DNA–topoisomerase II complex, DOX prevents the re-ligation of DNA strands, leading to persistent double-strand breaks that activate apoptotic pathways, thereby contributing to tumor cell death (Figure 2). Topoisomerase II is crucial in DNA replication and repair, and its inhibition by DOX disrupts these processes, leading to cell death [58,59].

Additionally, DOX induces oxidative stress via ROS generation. The quinone moiety of DOX undergoes redox cycling within mitochondria, where it is reduced by mitochondrial complex I to a semiquinone radical that transfers electrons to molecular oxygen, generating superoxide anions and hydrogen peroxide [47]. This excessive accumulation of ROS damages different cellular macromolecules, such as lipids, proteins, and nucleic acids, thus enhancing the cytotoxic effects of the drug [60]. However, this oxidative stress is a double-edged sword; it enhances DOX’s efficacy against tumors and underlies its dose-dependent cardiotoxicity. The cardiotoxic effects of DOX arise from mitochondrial dysfunction, disruption of calcium homeostasis, and apoptotic signaling, ultimately leading to irreversible cardiomyopathy and heart failure [60,61]. Moreover, the metabolic transformation of DOX into its major metabolite, doxorubicinol (DOXol), exacerbates cardiac damage by impairing mitochondrial ATP production and altering calcium homeostasis, further amplifying its cardiotoxic potential [56,62,63].

### 2.2. Clinical Challenges: Cardiotoxicity and Multidrug Resistance

Cardiotoxicity poses a significant limitation to the clinical use of DOX, affecting approximately 11% of patients undergoing treatment. The severity of cardiac dysfunction correlates strongly with cumulative DOX exposure, and the prognosis is often poor once congestive heart failure develops, with mortality rates nearing 50% [9,64]. The mechanisms underlying DOX-induced cardiotoxicity include mitochondrial oxidative stress, lipid peroxidation, and damage to cardiac myofibrils [65]. Various strategies have been explored to mitigate this adverse effect. These include using cardioprotective agents such as dexrazoxane, which chelates iron and reduces oxidative stress, and modifying drug delivery systems to enhance tumor targeting while sparing normal tissues [66]. Other strategies involve using antioxidants to counteract oxidative stress and developing novel drug formulations that release DOX in a controlled manner to reduce its systemic exposure.

Emerging from the clinical landscape is the challenge of drug resistance, which significantly limits the effectiveness of DOX-based chemotherapy. A primary resistance mechanism involves the overexpression of efflux transporters, notably P-glycoprotein (P-gp). These transporters actively extrude DOX from cancer cells, reducing intracellular drug concentrations and diminishing therapeutic efficacy [67]. In addition to mediating resistance, MDR mechanisms like P-gp overexpression may indirectly contribute to DOX-induced toxicity by modulating oxidative stress responses [68,69]. By reducing intracellular DOX accumulation, these efflux pumps alter the balance of ROS production and antioxidant defenses, potentially exacerbating systemic toxicity, including cardiotoxic effects. Furthermore, metabolic adaptations such as increased activity of aldo-keto reductases promote DOXol formation, compounding cardiac injury [70,71].

Beyond P-gp-mediated efflux, the tumor microenvironment plays a pivotal role in DOX resistance [72]. Hypoxic conditions, extracellular matrix remodeling, and interactions with stromal components contribute to diminished drug penetration and enhanced cell survival signaling [73]. Hypoxia-inducible factors (HIFs) regulate the expression of pro-survival genes, while cancer-associated fibroblasts (CAFs) secrete protective cytokines that shield tumor cells from DOX-induced apoptosis [74]. These factors collectively contribute to tumor persistence and necessitate novel strategies to enhance DOX efficacy [75]. Understanding and targeting the tumor microenvironment is crucial in overcoming drug resistance and improving the effectiveness of DOX-based chemotherapy.

### 2.3. Chemotherapeutic Applications and Combination Therapies

While DOX remains a fundamental component of many cancer treatment regimens, its dose-dependent cardiotoxicity significantly limits its clinical application. Identifying high-risk patients and implementing cardioprotective measures are essential to improving treatment safety. Adjusting the cumulative dose based on patient-specific risk factors helps reduce the likelihood of severe cardiotoxicity while maintaining therapeutic efficacy [76,77,78]. Recent studies have explored cardioprotective strategies to mitigate the off-target toxicity of DOX. Notably, a small-molecule allosteric inhibitor of BAX protein has been shown to protect against DOX-induced cardiomyopathy by inhibiting mitochondrial-mediated apoptosis pathways [78]. BAX is a pro-apoptotic protein that facilitates apoptosis by inducing permeabilization of the mitochondrial outer membrane, a key event in the programmed cell death pathway [79]. Similarly, dexrazoxane, an FDA-approved cardioprotective agent, has been shown to reduce DOX-induced oxidative stress and DNA damage, thereby preserving cardiac function without compromising the drug’s anticancer efficacy [80].

In combination therapies, DOX enhances therapeutic outcomes, mitigates drug resistance, and improves patient survival rates. It is often used alongside agents such as ascorbic acid, cinobufagin, cyclophosphamide, fluorouracil, or taxanes [81,82,83,84]. These combinations boost cytotoxicity against cancer cells and help overcome multidrug resistance (MDR). For example, in hematological malignancies such as non-Hodgkin’s lymphoma, DOX is a key component of the CHOP regimen (chemotherapy using a combination of drugs including cyclophosphamide, DOX, vincristine, and prednisone), which effectively induces remission [85]. In Hodgkin’s lymphoma, DOX is included in the ABVD regimen (chemotherapy using a combination of drugs including adriamycin, bleomycin, vinblastine, and dacarbazine), significantly improving response rates and survival.

The combination of chemotherapy with immunotherapy is another effective treatment strategy that offers synergistic effects and the potential to overcome drug resistance. DOX has been shown to upregulate PD-L1 expression on tumor and immune cells, contributing to an immunosuppressive tumor microenvironment that can be reversed with anti–PD-1/PD-L1 antibodies, thereby restoring cytotoxic T-cell activity and enhancing tumor regression, as seen in osteosarcoma and melanoma models [86]. For example, PD-L1–targeted DOX-loaded immunoliposomes in melanoma-bearing mice, induced full tumor regression in 20% of the animals, enhanced activated cytotoxic T-lymphocyte infiltration, and significantly improved survival compared to non-targeted liposomes and free DOX [87]. In parallel, PARP inhibitors like olaparib have shown strong synergy with DOX by impairing DNA repair, increasing apoptosis, and allowing for lower DOX doses without compromising efficacy in preclinical osteosarcoma studies [88]. Together, these strategies leverage both immune activation and DNA damage enhancement, potentially allowing lower DOX dosing to reduce toxicity while mitigating resistance mechanisms.

Despite these clinical and pre-clinical advances, DOX still faces limitations in achieving targeted delivery with minimal off-target toxicity. To address these challenges, ongoing research focuses on optimizing drug delivery systems, such as nanoparticle-based formulations, liposomal encapsulation, and targeted drug conjugates, to enhance efficacy while reducing adverse effects [89,90].

### 2.4. Innovations in Nanomedicine and Alternative Delivery Platforms

Recent advancements in nanomedicine have led to the exploration of novel delivery systems to enhance DOX’s safety and efficacy. Since the FDA approval of Doxil^®^, significant efforts have been dedicated to refining DOX’s liposomal formulation to improve its therapeutic outcomes. Enhanced PEGylation strategies have been developed to prolong circulation time and evade immune detection, while stealth liposomes have been engineered to bypass the mononuclear phagocyte system (MPS), leading to prolonged systemic retention and increased tumor accumulation via the EPR effect [91,92]. Furthermore, combining Doxil^®^ with immunotherapies, such as PD-1/PD-L1 checkpoint inhibitors, has shown promise in overcoming drug resistance. Clinical studies indicate that such combination approaches enhance immune responses while maintaining a favorable safety profile, reinforcing Doxil^®^’s role as a critical nanotherapeutic agent in oncology [14,93,94].

**Figure 3:**
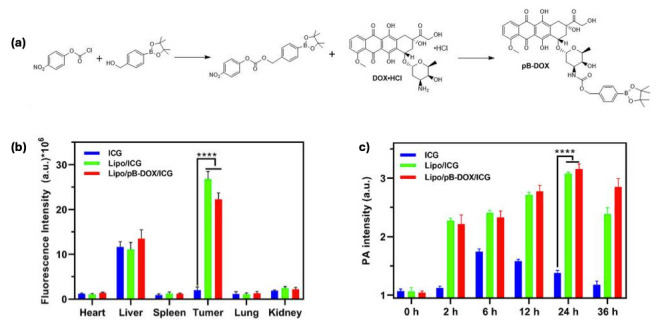
(**a**) Reaction scheme for the synthesis of DOX prodrug; (**b**) Semiquantitative biodistribution of free ICG, liposome containing ICG (Lipo/ICG), and liposome co-encapsulated with DOX prodrug and ICG (Lipo/pB-DOX/ICG); (**c**) Photoacoustic images of tumor sites on MDA-MB-231-tumor-bearing mice post-injection of free ICG, Lipo/ICG, and Lipo/pB-DOX/ICG at 2, 6, 12, 24, and 36 h. The data are shown as mean ± SD, n = 3 per group, **** *p* < 0.0001 [96]. Reprinted with permission from Ref. [96]. Copyright 2021, copyright Zhou et al.

A notable innovation is the emergence of stimuli-responsive liposomes designed for controlled drug release upon exposure to specific intracellular or extracellular triggers, such as pH shifts, enzymatic activity, temperature variations, ultrasound, or light [95]. These formulations enable site-specific drug delivery, reducing systemic toxicity and improving therapeutic precision. For example, indocyanine green, a well-known photosensitizer and ROS trigger, was conjugated to DOX to create a ROS-responsive DOX prodrug. This prodrug was subsequently encapsulated in a liposomal formulation to develop a combination therapy nanosystem. Upon irradiation, this nanosystem demonstrated significant therapeutic efficacy, achieving 94.5% tumor growth inhibition in MDA-MB-231 tumor-bearing mice compared to the control group (Figure 3) [96].

Myocet^®^, a non-PEGylated liposomal DOX formulation, has demonstrated clinical benefits, particularly for metastatic breast cancer in combination with cyclophosphamide [97]. This formulation is associated with reduced incidences of nausea, stomatitis, and vomiting compared to conventional DOX therapies. Additionally, it exhibits significantly lower cardiotoxicity due to its controlled drug release and selective tumor accumulation, making it a viable alternative to standard DOX therapy [98].

Despite these promising outcomes, liposomal DOX formulations continue to face challenges such as suboptimal drug retention and premature leakage [99,100,101]. One approach to improve their stability involves the use of photopolymerizable lipids that, upon light activation, form crosslinked polymer networks within the liposomal bilayer. This crosslinking reinforces the membrane, enhancing both its mechanical strength and its ability to retain lipophilic drugs. For example, hybrid liposomes incorporating photo-sensitive lipids have been shown to maintain their structural integrity and improve drug retention under physiological conditions, offering a more robust platform for therapeutic delivery [102]. Other approaches including remote loading techniques utilizing pH or ion gradients have been employed to enhance drug encapsulation efficiency and retention. However, these methods often pose limitations regarding scalability, stability, and reproducibility, which hinder their broader clinical translation [103,104,105,106]. Continued research into optimizing liposomal drug delivery platforms remains critical for advancing the clinical potential of nanomedicine-based DOX therapies.

Nanobubble-based drug delivery systems offer promising potential, particularly in ultrasound-triggered DOX release. These carriers enable site-specific drug delivery, reducing systemic toxicity. However, high drug loading can destabilize nanobubbles, compromising their clinical efficacy. Achieving an optimal balance between drug payload and carrier stability remains a key focus [107]. Another approach is to use magnetic nanoparticles (MNPs) for site-specific delivery, leveraging external magnetic fields to direct DOX accumulation at tumor sites [108]. Studies report high drug-loading efficiencies, with capacities reaching up to 870 μg of DOX per mg of MNPs [109]. However, ensuring uniform drug distribution and preventing nanoparticle aggregation in biological fluids pose significant challenges. Maintaining biocompatibility and stability while scaling up production remains a priority in MNP-based drug delivery research [110].

Graphene-based nanocarriers have garnered interest due to their high surface area, which allows DOX loading ratios exceeding 200% [111,112]. Despite this advantage, concerns regarding biocompatibility, toxicity, and biodegradability hinder clinical translation. Surface modifications with biopolymers are being explored to enhance their safety, but further research is needed to confirm their therapeutic suitability. Ensuring the safe degradation and elimination of graphene-based carriers is essential before they can be considered viable for DOX delivery [111,113].

## 3. Advancements in Drug Delivery Systems: Polymeric Nanocarriers

**Figure 4:**
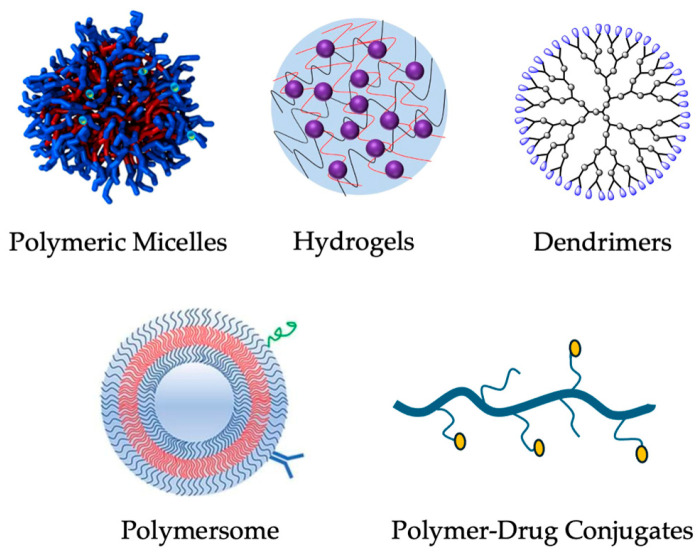
Illustration of common polymeric nanocarrier types: micelles, polymersomes, hydrogels, dendrimers, and polymer–drug conjugates [139,140,141]. Reprinted with permission from Ref. [139]. 2023, Elçin et al., Reprinted with permission from Ref. [140]. 2019, Ekladious et al. and Ref. [141]. 2022, Del Borgo et al.

To overcome the limitations of traditional DOX formulations, novel drug delivery strategies have been developed to improve the therapeutic index while minimizing toxicity. Among these, polymeric nanocarriers have garnered significant attention for their ability to provide controlled and sustained drug release, thereby reducing dosing frequency and enhancing drug bioavailability at the tumor site [114,115,116,117]. By incorporating polymers such as PLGA (poly(lactic-co-glycolic acid)) or PEG (polyethylene glycol), these nanoparticles can be tailored to enhance stability, biocompatibility, and even tumor targeting through surface modifications that recognize tumor-specific biomarkers. These polymeric nanocarriers, owing to their tunable properties, offer promising platforms for targeted and controlled release of anticancer agents [118,119,120,121]. Since different polymeric nanocarriers offer unique advantages, several types have been explored for DOX delivery (Figure 4), including polymeric micelles [122,123,124,125], polymer–drug conjugates [126,127], hydrogels [128,129,130,131], polymersomes [132,133], and dendrimers [134]. Polymeric micelles are extensively studied in breast cancers, with clinical trials demonstrating a ~58% overall response rate in metastatic breast cancer compared to conventional formulations [135,136]. Hydrogels are especially advantageous for localized drug delivery, allowing for targeted treatment while reducing systemic side effects. Moreover, they are widely employed in 3D cell culture systems for cancer modeling and high-throughput drug screening applications [137]. On the other hand, polymeric drug conjugates are effective for solid tumors like lung, breast, and ovarian cancers, as linking drugs to polymers such as PEG or polyglutamic acid improves circulation time and enables controlled release at tumor sites [138]. Similarly, each drug delivery system presents distinct structural attributes, advantages, and limitations, underscoring their specific roles and potential in oncological applications. These nanocarriers have shown variable efficacy depending on the cancer type and delivery strategy.

### 3.1. Polymeric Micelles

Polymeric micelles are self-assembled nanostructures formed by amphiphilic polymers in aqueous environments [142,143]. Their core-shell architecture (Figure 1) enables the encapsulation of hydrophobic drugs such as DOX within the core, enhancing the solubility and bioavailability of the encapsulated drug [122,144]. The successful performance of polymeric micelles is highly dependent on the physiochemical properties of the encapsulated drug. The hydrophobic core of the micelle serves as the primary site for drug loading, where interactions such as hydrophobic forces, π–π stacking, and hydrogen bonding between the core and the drug enhance therapeutic efficacy. Current micellar systems commonly utilize biocompatible and biodegradable polymers, such as polyesters and polyamides, as the hydrophobic domain. These systems are specifically designed to improve the solubility of lipophilic drugs in aqueous environments by leveraging the hydrophobic interactions between the drug and the polymer. However, most existing micellar formulations require five to ten times the polymer mass to encapsulate a given drug mass, primarily due to their low drug-loading capacity. As a result, higher amounts of excipients are needed to achieve the desired therapeutic drug concentration, leading to increased medication costs and potential side effects. Enhancing the drug-loading capacity of micellar systems could significantly improve therapeutic efficacy by delivering higher drug concentrations to tumor sites while reducing excipient-related toxicity and manufacturing expenses [145,146,147,148].

Conventional micellar systems primarily utilize hydrophobic interactions between the polymer and drug to enhance DLC [145,146,147]. This is mainly achieved by increasing the hydrophobic content in the polymer chain, which in turn reduces the stability of the micelles and can lead to premature leakage of the drug. An ideal micellar drug delivery system should have a balance between the hydrophobic and hydrophilic content to enhance the loading capacity without compromising its stability. This can be done by improving the interaction of the drug and the hydrophobic content of the polymer without altering their balance. Incorporating several non-covalent interactions such as electrostatic interaction, dipole–dipole interaction, hydrogen bonding, and π–π stacking along with the hydrophobic effect are shown to increase the loading capacity of the micellar carrier [145,149,150,151].

Several approaches have been reported to enhance the drug-loading capacity of the micellar systems. The easiest way to attain π–π interaction is to incorporate aromatic rings into the polymer backbone [152]. For example, Gu et.al conjugated cinnamate moiety in the hydrophobic core of the micelles to demonstrate π–π interaction and the stability after drug encapsulation. Interestingly, when they doubled the cinnamate group in the micelles, a two-fold increase in the DLC was observed. Additionally, the increment in the cinnamate group also showed lower critical micellar concentration values and better anticancer activity in vitro [153].

**Figure 5:**
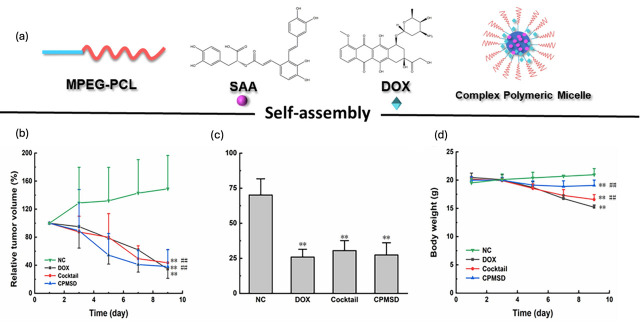
(**a**) Co-loading of DOX and polyphenol within PCL-based polymeric micelles; Evaluation of anticancer efficacy of various preparations in nude mice bearing human breast cancer MCF-7 cells (n = 8, mean ± SD) by relative tumor volume (**b**), tumor weight (**c**), and body weight change with time (**d**) [155]. ** *p* < 0.01 compared with the NC group and ## *p* < 0.01 compared with DOX group, Reprinted with permission from Ref. [155]. 2022, Xu.

The enhancement in DLC through π–π interactions has been further validated by similar studies. For example, Zhang et al. reported that modifying the hydrophobic block of amphiphilic copolymers with aromatic groups significantly increased the DLC, achieving up to 18% DOX loading within the micelles [154]. In a different approach, Li et al. combined DOX with a polyphenol, salvianolic acid, for breast cancer chemotherapy [155]. The study employed Design of Experiments (DOE) to optimize the micellar formulation, identifying the optimal DOX-to-carrier ratio as 1:5 and the DOX-to-salvianolic acid ratio as 1:4. This optimization yielded a high DLC of 15.7 ± 0.8% and an encapsulation efficiency (EE) exceeding 95%, closely matching the DOE-predicted values of 14.3% for DLC and 94.4% for EE. Most importantly, the formulation maintained DOX’s anticancer efficacy while improving its cardioprotective effect against oxidative stress-induced injuries in tumor-bearing mice (Figure 5).

**Figure 6:**
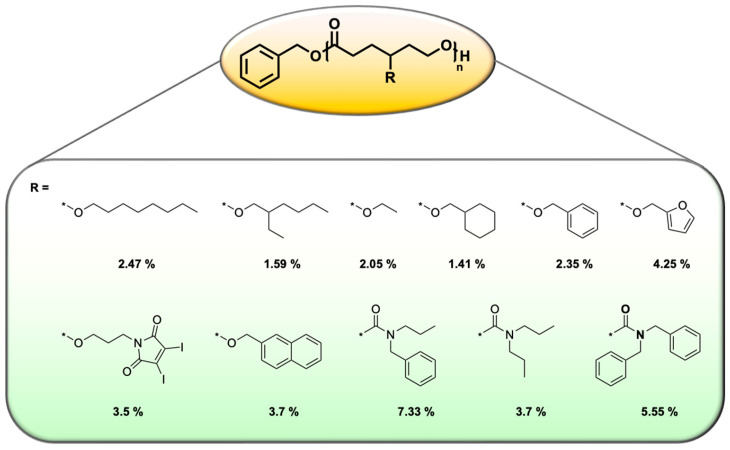
PCLs with different pendant groups and their corresponding DLC values [118,158,159,160,161].

Rececntly, many biodegradable polymers have been explored for drug delivery applications, with polycaprolactones (PCLs) emerging as prime candidates due to their synthetic versatility and ease of functionalization [156,157]. Incorporating various functional groups into PCLs has enabled a deeper understanding of the relationship between polymer structure and micelle properties. Functional groups such as alkyl, benzyl, maleimide, and furan have been introduced as pendant groups to enhance interactions with DOX through π–π stacking, hydrogen bonding, and hydrophobic interactions. These modifications have significantly improved DLC in several studies, increasing the DLC from 1.41% to 7.33% (Figure 6) [118,142,158,159,160,161]. This enhancement is particularly noteworthy, as PCL-based systems typically exhibit poor DOX loading. Furthermore, co-loading DOX with polyphenols such as resveratrol and quercetin in PCL-based systems has also enhanced DLC [162,163]. For example, PEG-*b*-PBCL micelles co-loaded with resveratrol and quercetin achieved DLCs of 8.7% and 10%, respectively, demonstrating the synergistic effect of polyphenol co-loading on drug incorporation.

Polymeric micelles are often engineered for passive and/or active targeting to improve selection, thereby minimizing drug side effects. Passive targeting primarily relies on the EPR [161] effect, which allows nanoscale carriers (typically 10–200 nm in size) to preferentially accumulate in tumor tissues due to their leaky vasculature and poor lymphatic drainage [164,165,166]. For example, Stefan et al. developed several polycaprolactone-based micellar systems, including LA-PCL, PBACL, and PME3DDCL, with particle sizes optimized within this range to achieve efficient passive tumor targeting [120,167,168]. In parallel, active targeting strategies involve surface modification of micelles with ligands that can selectively bind to overexpressed receptors on cancer cell membranes. This approach enhances cellular uptake and improves drug specificity [156,169]. A study by Nasr et al. demonstrated this approach, where folic acid was conjugated to Tamoxifen citrate micelles to target folate receptors, which are commonly overexpressed in certain cancer types such as breast cancer. The folate-functionalized micelles demonstrated significantly improved tumor cell specificity and drug delivery efficiency, underscoring the potential of ligand-mediated active targeting in cancer therapy [170].

### 3.2. Hydrogels

Hydrogels are 3D, hydrophilic polymer networks capable of forming a gel in situ by absorbing substantial amounts of water. Since they are often administered directly to site-specific tissues, they can offer controlled and sustained drug release at the target location, thereby minimizing systemic drug exposure. Physically crosslinked systems are often preferred, as they eliminate the need for photoirradiation, organic solvents, and catalysts. To create a reusable hydrogel, An et al. designed a self-healing biodegradable pectin-based hydrogel composed of P(NIPAM-*stat*-AH)/pectin-CHO and acylhydrazide functionalized polymer poly(N-isopropylacrylamide-stat-acylhydrazide) P(NIPAM-*stat*-AH) to deliver DOX into the tumor [171]. Due to the dynamic acylhydrazone bonds, the hydrogel exhibits self-healing properties with tunable mechanical strength based on the P(NIPAM-*stat*-AH) to pectin-CHO ratio. In vitro and in vivo studies confirmed its biocompatibility, biodegradability, reduced drug toxicity, and controlled release, supporting its potential as a synergistic anti-tumor drug delivery system.

Despite their ability to provide controlled drug release, most hydrogels still exhibit low DLC, particularly when encapsulating DOX [172]. Alipournazari et al. synthesized a starch/PVA/g-C_3_N_4_-based hydrogel and evaluated its potential for DOX loading in breast cancer treatment, achieving a drug loading efficiency of 44.75% and an entrapment efficiency of 88% [173]. To further enhance DLC, newer polymer systems have been developed. Notably, a recent study reported the synthesis of a chitosan and poly(acrylamide-co-maleic acid) blend, termed chitosan-poly(acrylamide-maleic acid) (Ch-p(Ac-Ma)), which achieved a DLC exceeding 90% for DOX [174]. Moreover, the favorable binding affinity observed in molecular docking simulations (−8.3 kcal/mol) further supports its potential as an effective HER2-targeting anticancer agent, in agreement with its known experimental activity.

### 3.3. Dendrimers

**Figure 7:**
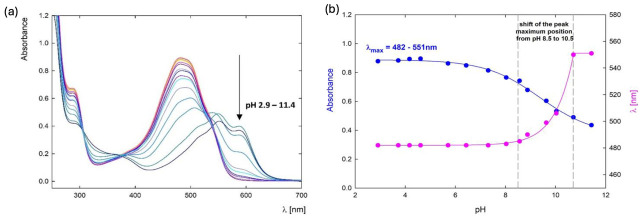
(**a**) UV–Vis spectra of DOX in water (50 ppm) in the pH range of 2.9–11.4; (**b**) The position of the absorbance maximum of the UV–Vis spectra for DOX (blue curve) and the shift of the absorbance maximum depending on the pH of the solution (pink curve). Reprinted with permission from Ref. [178]. 2023, copyright Szota et al.

Dendrimers are highly branched, tree-like polymers with a central core, internal branches, and terminal functional groups. Their unique architecture allows for precise control over particle size and surface functionality [175]. PAMAM (polyamidoamine) dendrimers conjugated with methotrexate have demonstrated enhanced targeting and cytotoxicity towards cancer cells, owing to their multivalency and ability to facilitate drug uptake. However, their synthesis is complex, leading to high production costs. Moreover, prolonged administration of PAMAM dendrimers can cause organ and tissue toxicities [176]. Soltany et al. recently reported a folic acid conjugated poly (amidoamine) dendrimer (FA–PAMAMG2–MCS), which achieved a maximum DOX adsorption capacity of 102.85 mg g^−1^. This dendrimer was explored for its active targeting capacity due to the presence of the folate on the dendrimer surface, which can actively target cancer cells with over-expressed folate receptors to drastically increase the selectivity of the nanocarrier [177]. Similarly to other polymeric nanocarriers, the DLC of DOX in dendrimers can be optimized by adjusting the polymer-to-DOX ratio. Another interesting study reported by Szota et al. demonstrated that the DLC of DOX is highly dependent on the pH environment (Figure 7). The DLC of DOX increased as the pH decreased—pH values of 9, 9.5, and 10 yielded DLCs of 8.4%, 7.6%, and 4.8%, respectively. In addition, the highest DLC (39.2%) was achieved with a polymer-to-DOX ratio of 1:24 at a pH of 9.5 [178]. This pH sensitivity could be advantageous in targeting the acidic tumor microenvironment, potentially increasing drug release at the site of action.

### 3.4. Polymersomes

Polymerosmes are vesicular structures formed by the self-assembly of amphiphilic polymers, similarly to liposomes, but with bilayer polymer membranes that offer greater stability and reduced premature drug leakage. Their bilayer makes them interesting for exploring encapsulating hydrophilic and hydrophobic drugs for combinational therapy. However, polymersome synthesis is complex, requires price control, and is often associated with low DLC [179]. For example, Ferrero et al. synthesized mPEG–PDH–mPEG polymersomes, achieving a DLC of 9.8 wt.% and an impressive drug-loading efficiency (DLE) of 98 wt.%, significantly surpassing most conventional polymeric nanocarriers [180]. Other studies have demonstrated the potential of polymersomes to co-encapsulate multiple anticancer agents such as Docetaxel, Rapamycin, and Afatinib, reaching a combined DLC of up to 20 wt.% [179]. To improve the DLC of polymersomes, poly(ethylene glycol)–polycaprolactone–poly(ethylene glycol) [PEG-PCL-SS-PCL-PEG], a triblock copolymer that self-assembles into monodispersed polymersomes, was synthesized and its ability to load DOX explored. Due to the high hydrophobic chain, a high DLC and encapsulation efficiency of 16.13 ± 1.05% and 60.74 ± 3.95%, respectively, were reported [181].

### 3.5. Polymeric Drug Conjugates

Polymeric drug conjugates involve the covalent attachment of therapeutic agents to polymers, enhancing solubility and stability. A prominent example is the conjugation of DOX to N-(2-hydroxypropyl)methacrylamide (HPMA) copolymers [182,183]. This conjugate exhibited reduced cardiotoxicity and enhanced accumulation in tumor tissues, leading to improved therapeutic outcomes. This strategy has been adopted to increase the DLC of DOX. For example, Braunová et al. synthesized a series of star-like amphiphilic polymer-DOX conjugates, termed FP1-Dox–FP4-Dox. The FP1-DOX had the highest DLC of 10.7%, resulting in the highest cancer cell death among the synthesized series [184]. In a similar study, Ou et al. synthesized *N*-(2-hydroxypropyl) methacrylamide (HPMA) polymer–DOX with DOX encapsulation of 7.1 *w*/*w*%. Interestingly, this polymer–DOX conjugate exhibited longer conjugate half-life with no cardiotoxicity in micelles [185]. Aside from the numerous advantages of polymeric drug conjugates, it is imperative to note that the covalent bonding can alter the drug’s activity, and the conjugates may face challenges in achieving efficient intracellular drug release as a result of the strong covalent bond between the drug and the polymer [138,186].

In recent decades, several strategies have been developed to incorporate stimuli-responsive linkers that enable the site-specific release of doxorubicin (DOX) from polymeric drug conjugates. These include pH-sensitive linkers (hydrazone or Schiff-base bonds), which cleave in the acidic environments of tumors or endosomes, allowing for selective intracellular drug release. Redox-responsive linkers, such as disulfide bonds, take advantage of elevated intracellular glutathione levels in cancer cells to trigger drug release. Additionally, enzyme-cleavable peptide linkers, including cathepsin B- or matrix metalloproteinase (MMP)-sensitive sequences, provide tumor-specific activation [187]. Emerging approaches, such as coiled-coil peptides and dynamic covalent linkers, offer reversible, environmentally responsive mechanisms for enhanced control over drug release [188,189]. Collectively, these strategies improve the therapeutic precision and efficacy of polymeric drug conjugates while minimizing off-target toxicity.

### 3.6. Clinical Challenges

Despite their potential, polymeric nanocarriers face significant clinical translation barriers, primarily due to their unpredictable pharmacokinetic behavior in vivo. One major challenge is maintaining structural stability in the bloodstream. Nanocarriers must remain intact during circulation to prevent premature drug leakage, which compromises tumor targeting and increases off-target toxicity. However, their stability can be affected by dilution, enzymatic degradation, or non-specific interactions with serum components, necessitating careful optimization of polymer chemistry and architecture [190,191]. To prolong circulation half-life, polymer surfaces are often PEGylated, which reduces protein adsorption and opsonization. While effective, the success of PEGylation depends on PEG chain length, density, and conformational flexibility. Previous reports have shown that dense PEG coatings on block copolymer nanoparticles increased plasma retention from 10% to 85% of the injected dose after 4 h [192]. However, this strategy is not without drawbacks. Repeated administration of PEGylated nanoparticles can induce anti-PEG antibodies, triggering an accelerated blood clearance (ABC) phenomenon, thus undermining long-term therapeutic use [193]. Another critical factor is the formation of a protein corona, a layer of plasma proteins that adsorbs onto nanoparticle surfaces immediately upon systemic exposure. This corona significantly influences biodistribution, targeting efficiency, and clearance rates. If the corona is rich in opsonins (IgG, complement proteins), it promotes recognition and rapid clearance by the mononuclear phagocyte system (MPS). In contrast, enrichment with dysopsonins such as albumin or apolipoproteins may prolong circulation [194]. The composition of the protein corona is dynamic and patient-specific, contributing to the variability and unpredictability of clinical outcomes for polymer-based drug delivery systems [195].

## 4. Future Directions in Doxorubicin Delivery Systems

The integration of nanotechnology continues to revolutionize DOX delivery. Liposomal formulations such as Doxil^®^, Caelyx^®^, Myocet^®^, and Lipo-dox^®^ have significantly improved pharmacokinetics and reduced toxicity compared to traditional DOX. Despite significant advancements in DOX delivery systems, challenges persist, such as limited cellular uptake of DOX in tumor tissues and slow drug release from liposomal formulations, which may contribute to adverse effects such as palmar plantar erythrodysesthesia. Researchers are exploring polymeric nanocarriers as alternative drug delivery strategies to address these issues. Clinical trials of formulations like SP1049C (Phase III) and NK911 (Phase II) have shown improved drug delivery profiles. Additionally, polymeric conjugates such as FCE28068/PK1, currently in Phase II trials, offer enhanced pharmacokinetics and selective tumor targeting.

Future advancements aim to synergize DOX with immunotherapies and develop next-generation nanocarriers with superior drug-loading capacity, stability, and selective tumor targeting. Combining DOX with vascular-disrupting agents (VDAs) like DMXAA has shown promise in enhancing antitumor immune responses by disrupting tumor vasculature while activating immune cells. Moreover, fluorescence probe-tagged nanocarriers enable simultaneous drug delivery and real-time cellular imaging, improving treatment precision. Ongoing research into these novel formulations will continue to refine DOX’s applications, paving the way for safer and more effective cancer treatments.

## 5. Conclusions

Given the widespread application of DOX in chemotherapy and its use in combination therapies, extensive efforts have been made to mitigate its side effects while enhancing its therapeutic efficacy. Strategies such as cardioprotective agents and advanced formulation techniques have been explored to reduce toxicity and improve drug retention. In particular, innovations in nanomedicine have led to the development of alternative drug delivery platforms, including polymeric nanocarriers, which enhance DOX loading capacity, prolong circulation time, and improve tumor targeting. Polymeric micelles, dendrimers, polymersomes, hydrogels, and polymeric drug conjugates have emerged as promising carriers for DOX delivery, aiming to optimize its pharmacokinetics and biodistribution while minimizing systemic toxicity. As research in this field advances, future directions will focus on refining these delivery systems to further improve the stability and targeted release, ultimately improving the safety and efficacy of DOX-based cancer treatments.

## Data Availability

Not applicable.

## References

[1] Siegel R.L., Kratzer T.B., Giaquinto A.N., Sung H., Jemal A. (2025). Cancer statistics, 2025. CA Cancer J. Clin..

[2] Chen H., Khemtong C., Yang X., Chang X., Gao J. (2011). Nanonization strategies for poorly water-soluble drugs. Drug Discov. Today.

[3] Senapati S., Mahanta A.K., Kumar S., Maiti P. (2018). Controlled drug delivery vehicles for cancer treatment and their performance. Signal Transduct. Target. Ther..

[4] Johnson-Arbor K., Dubey R. (2025). Doxorubicin. StatPearls.

[5] Thorn C.F., Oshiro C., Marsh S., Hernandez-Boussard T., McLeod H., Klein T.E., Altman R.B. (2011). Doxorubicin pathways: Pharmacodynamics and adverse effects. Pharmacogenet. Genom..

[6] Carvalho C., Santos R.X., Cardoso S., Correia S., Oliveira P.J., Santos M.S., Moreira P.I. (2009). Doxorubicin: The good, the bad and the ugly effect. Curr. Med. Chem..

[7] Khasraw M., Bell R., Dang C. (2012). Epirubicin: Is it like doxorubicin in breast cancer? A clinical review. Breast.

[8] Ma X., Sun R., Cheng J., Liu J., Gou F., Xiang H., Zhou X. (2016). Fluorescence aggregation-caused quenching versus aggregation-induced emission: A visual teaching technology for undergraduate chemistry students. J. Chem. Educ..

[9] Swain S.M., Whaley F.S., Ewer M.S. (2003). Congestive heart failure in patients treated with doxorubicin: A retrospective analysis of three trials. Cancer.

[10] Swain S.M., Whaley F.S., Gerber M.C., Weisberg S., York M., Spicer D., Jones S.E., Wadler S., Desai A., Vogel C. (1997). Cardioprotection with dexrazoxane for doxorubicin-containing therapy in advanced breast cancer. J. Clin. Oncol..

[11] Afsar T., Razak S., Almajwal A., Al-Disi D. (2020). Doxorubicin-induced alterations in kidney functioning, oxidative stress, DNA damage, and renal tissue morphology; Improvement by *Acacia hydaspica* tannin-rich ethyl acetate fraction. Saudi J. Biol. Sci..

[12] Du J., Zhang A., Li J., Liu X., Wu S., Wang B., Wang Y., Jia H. (2021). Doxorubicin-Induced Cognitive Impairment: The Mechanistic Insights. Front. Oncol..

[13] Kamińska K., Cudnoch-Jędrzejewska A. (2023). A Review on the Neurotoxic Effects of Doxorubicin. Neurotox. Res..

[14] Barenholz Y. (2012). Doxil^®^—The first FDA-approved nano-drug: Lessons learned. J. Control. Release.

[15] Matsumura Y., Maeda H. (1986). A new concept for macromolecular therapeutics in cancer chemotherapy: Mechanism of tumoritropic accumulation of proteins and the antitumor agent smancs. Cancer Res..

[16] Liechty W.B., Kryscio D.R., Slaughter B.V., Peppas N.A. (2010). Polymers for drug delivery systems. Annu. Rev. Chem. Biomol. Eng..

[17] Sung Y.K., Kim S.W. (2020). Recent advances in polymeric drug delivery systems. Biomater. Res..

[18] Venditti I. (2019). Morphologies and functionalities of polymeric nanocarriers as chemical tools for drug delivery: A review. J. King Saud Univ. Sci..

[19] Cabral H., Miyata K., Osada K., Kataoka K. (2018). Block copolymer micelles in nanomedicine applications. Chem. Rev..

[20] Peer D., Karp J.M., Hong S., Farokhzad O.C., Margalit R., Langer R. (2007). Nanocarriers as an emerging platform for cancer therapy. Nat. Nanotechnol..

[21] Davis M.E., Chen Z., Shin D.M. (2010). Nanoparticle therapeutics: An emerging treatment modality for cancer. Nat. Rev. Drug Discov..

[22] Lammers T., Hennink W., Storm G. (2008). Tumour-targeted nanomedicines: Principles and practice. Br. J. Cancer.

[23] Daglar B., Ozgur E., Corman M., Uzun L., Demirel G. (2014). Polymeric nanocarriers for expected nanomedicine: Current challenges and future prospects. RSC Adv..

[24] Min Y., Caster J.M., Eblan M.J., Wang A.Z. (2015). Clinical translation of nanomedicine. Chem. Rev..

[25] Cabral H., Kataoka K. (2014). Progress of drug-loaded polymeric micelles into clinical studies. J. Control. Release.

[26] Wicki A., Witzigmann D., Balasubramanian V., Huwyler J. (2015). Nanomedicine in cancer therapy: Challenges, opportunities, and clinical applications. J. Control. Release.

[27] McCarthy T.D., Karellas P., Henderson S.A., Giannis M., O’Keefe D.F., Heery G., Paull J.R., Matthews B.R., Holan G. (2005). Dendrimers as drugs: Discovery and preclinical and clinical development of dendrimer-based microbicides for HIV and STI prevention. Mol. Pharm..

[28] Ghosh R., Malhotra M., Sathe R.R.M., Jayakannan M. (2020). Biodegradable polymer theranostic fluorescent nanoprobe for direct visualization and quantitative determination of antimicrobial activity. Biomacromolecules.

[29] Svenson S. (2015). The dendrimer paradox–high medical expectations but poor clinical translation. Chem. Soc. Rev..

[30] Kakkar A., Traverso G., Farokhzad O.C., Weissleder R., Langer R. (2017). Evolution of macromolecular complexity in drug delivery systems. Nat. Rev. Chem..

[31] Prabhakar U., Maeda H., Jain R.K., Sevick-Muraca E.M., Zamboni W., Farokhzad O.C., Barry S.T., Gabizon A., Grodzinski P., Blakey D.C. (2013). Challenges and key considerations of the enhanced permeability and retention effect for nanomedicine drug delivery in oncology. Cancer Res..

[32] Yan S., Na J., Liu X., Wu P. (2024). Different Targeting Ligands-Mediated Drug Delivery Systems for Tumor Therapy. Pharmaceutics.

[33] Liu M., Fang X., Yang Y., Wang C. (2021). Peptide-Enabled Targeted Delivery Systems for Therapeutic Applications. Front. Bioeng. Biotechnol..

[34] Ferris R.L., Jaffee E.M., Ferrone S. (2010). Tumor antigen-targeted, monoclonal antibody-based immunotherapy: Clinical response, cellular immunity, and immunoescape. J. Clin. Oncol..

[35] Khan S., Hussain A., Fahimi H., Aliakbari F., Haj Bloukh S., Edis Z., Mahdi Nejadi Babadaei M., Izadi Z., Shiri Varnamkhasti B., Jahanshahi F. (2022). A review on the therapeutic applications of aptamers and aptamer-conjugated nanoparticles in cancer, inflammatory and viral diseases. Arab. J. Chem..

[36] Gaucher G., Dufresne M.-H., Sant V.P., Kang N., Maysinger D., Leroux J.-C. (2005). Block copolymer micelles: Preparation, characterization and application in drug delivery. J. Control. Release.

[37] Huang Y., Yan J., Peng S., Tang Z., Tan C., Ling J., Lin W., Lin X., Zu X., Yi G. (2020). pH/Reduction Dual-Stimuli-Responsive Cross-Linked Micelles Based on Multi-Functional Amphiphilic Star Copolymer: Synthesis and Controlled Anti-Cancer Drug Release. Polymers.

[38] Li S., Wu W., Xiu K., Xu F., Li Z., Li J. (2014). Doxorubicin loaded pH-responsive micelles capable of rapid intracellular drug release for potential tumor therapy. J. Biomed. Nanotechnol..

[39] Yang C., Xiao J., Xiao W., Lin W., Chen J., Chen Q., Zhang L., Zhang C., Guo J. (2017). Fabrication of PDEAEMA-based pH-responsive mixed micelles for application in controlled doxorubicin release. RSC Adv..

[40] Alibolandi M., Sadeghi F., Abnous K., Atyabi F., Ramezani M., Hadizadeh F. (2015). The chemotherapeutic potential of doxorubicin-loaded PEG-b-PLGA nanopolymersomes in mouse breast cancer model. Eur. J. Pharm. Biopharm..

[41] Miao L., Guo S., Zhang J., Kim W.Y., Huang L. (2014). Nanoparticles with Precise Ratiometric Co-Loading and Co-Delivery of Gemcitabine Monophosphate and Cisplatin for Treatment of Bladder Cancer. Adv. Funct. Mater..

[42] Sarniak A., Lipinska J., Tytman K., Lipinska S. (2016). Endogenous mechanisms of reactive oxygen species (ROS) generation. Postep. Hig. Med. Dosw..

[43] Mastrangelo S., Attina G., Triarico S., Romano A., Maurizi P., Ruggiero A. (2022). The DNA-topoisomerase inhibitors in cancer therapy. Biomed. Pharmacol. J..

[44] Sinha S.J., Kumar B., Prasad C.P., Chauhan S.S., Kumar M. (2025). Emerging Research and Future Directions on Doxorubicin: A Snapshot. Asian Pac. J. Cancer Prev..

[45] Arcamone F., Cassinelli G., Fantini G., Grein A., Orezzi P., Pol C., Spalla C. (1969). Adriamycin, 14-hydroxydaimomycin, a new antitumor antibiotic from S. Peucetius var. caesius. Biotechnol. Bioeng..

[46] Von Hoff D.D., Rozencweig M., Layard M., Slavik M., Muggia F.M. (1977). Daunomycin-induced cardiotoxicity in children and adults. A review of 110 cases. Am. J. Med..

[47] Wallace K.B., Sardão V.A., Oliveira P.J. (2020). Mitochondrial Determinants of Doxorubicin-Induced Cardiomyopathy. Circ. Res..

[48] Alrushaid S., Sayre C.L., Yáñez J.A., Forrest M.L., Senadheera S.N., Burczynski F.J., Löbenberg R., Davies N.M. (2017). Pharmacokinetic and Toxicodynamic Characterization of a Novel Doxorubicin Derivative. Pharmaceutics.

[49] Pushkaran A.C., Arabi A.A. (2025). Accurate prediction of DNA-Intercalator binding energies: Ensemble of short or long molecular dynamics simulations?. Int. J. Biol. Macromol..

[50] Lee J., Choi M.-K., Song I.-S. (2023). Recent Advances in Doxorubicin Formulation to Enhance Pharmacokinetics and Tumor Targeting. Pharmaceuticals.

[51] Thotakura N., Panjeta A., Negi P., Preet S., Raza K. (2021). Doxorubicin-Loaded Mixed Micelles for the Effective Management of Skin Carcinoma: In Vivo Anti-Tumor Activity and Biodistribution Studies. AAPS PharmSciTech.

[52] Imantay A., Mashurov N., Zhaisanbayeva B.A., Mun E.A. (2025). Doxorubicin-Conjugated Nanoparticles for Potential Use as Drug Delivery Systems. Nanomaterials.

[53] Bansal N., Adams M.J., Ganatra S., Colan S.D., Aggarwal S., Steiner R., Amdani S., Lipshultz E.R., Lipshultz S.E. (2019). Strategies to prevent anthracycline-induced cardiotoxicity in cancer survivors. Cardiooncology.

[54] Palvia A.R., Damera A.R., Nandi A.R., Magar S., Patidar S., Kasarla S., Ghantasala V., Shah M.K., Goyal M. (2024). Cardio-Oncology’s Modern Approaches to Prevent Doxorubicin-Induced Cardiotoxicity: A Systematic Review. Cureus.

[55] Alghamdi M.A., Fallica A.N., Virzi N., Kesharwani P., Pittala V., Greish K. (2022). The Promise of Nanotechnology in Personalized Medicine. J. Pers. Med..

[56] Linders A.N., Dias I.B., Lopez Fernandez T., Tocchetti C.G., Bomer N., Van der Meer P. (2024). A review of the pathophysiological mechanisms of doxorubicin-induced cardiotoxicity and aging. NPJ Aging.

[57] Pang B., Qiao X., Janssen L., Velds A., Groothuis T., Kerkhoven R., Nieuwland M., Ovaa H., Rottenberg S., van Tellingen O. (2013). Drug-induced histone eviction from open chromatin contributes to the chemotherapeutic effects of doxorubicin. Nat. Commun..

[58] Tacar O., Sriamornsak P., Dass C.R. (2013). Doxorubicin: An update on anticancer molecular action, toxicity and novel drug delivery systems. J. Pharm. Pharmacol..

[59] Pommier Y., Leo E., Zhang H., Marchand C. (2010). DNA topoisomerases and their poisoning by anticancer and antibacterial drugs. Chem. Biol..

[60] Doroshow J.H. (2019). Mechanisms of Anthracycline-Enhanced Reactive Oxygen Metabolism in Tumor Cells. Oxid. Med. Cell. Longev..

[61] Kciuk M., Gielecinska A., Mujwar S., Kolat D., Kaluzinska-Kolat Z., Celik I., Kontek R. (2023). Doxorubicin-An Agent with Multiple Mechanisms of Anticancer Activity. Cells.

[62] Solem L.E., Henry T.R., Wallace K.B. (1994). Disruption of mitochondrial calcium homeostasis following chronic doxorubicin administration. Toxicol. Appl. Pharmacol..

[63] Zhou S., Starkov A., Froberg M.K., Leino R.L., Wallace K.B. (2001). Cumulative and irreversible cardiac mitochondrial dysfunction induced by doxorubicin. Cancer Res..

[64] Haq M.M., Legha S.S., Choksi J., Hortobagyi G.N., Benjamin R.S., Ewer M., Ali M. (1985). Doxorubicin-induced congestive heart failure in adults. Cancer.

[65] Zhang S., Liu X., Bawa-Khalfe T., Lu L.S., Lyu Y.L., Liu L.F., Yeh E.T. (2012). Identification of the molecular basis of doxorubicin-induced cardiotoxicity. Nat. Med..

[66] Ghasemi K., Vaseghi G., Mansourian M. (2021). Pharmacological interventions for preventing anthracycline-induced clinical and subclinical cardiotoxicity: A network meta-analysis of metastatic breast cancer. J. Oncol. Pharm. Pract..

[67] Muzzammil T., Moore M.J., Hedley D., Ballinger J.R. (2001). Comparison of ^99m^Tc-sestamibi and doxorubicin to monitor inhibition of P-glycoprotein function. Br. J. Cancer.

[68] Shen F., Chu S., Bence A.K., Bailey B., Xue X., Erickson P.A., Montrose M.H., Beck W.T., Erickson L.C. (2008). Quantitation of doxorubicin uptake, efflux, and modulation of multidrug resistance (MDR) in MDR human cancer cells. J. Pharmacol. Exp. Ther..

[69] Shchulkin A.V., Abalenikhina Y.V., Kosmachevskaya O.V., Topunov A.F., Yakusheva E.N. (2024). Regulation of P-Glycoprotein during Oxidative Stress. Antioxidants.

[70] Matsunaga T., Kawabata S., Yanagihara Y., Kezuka C., Kato M., Morikawa Y., Endo S., Chen H., Iguchi K., Ikari A. (2019). Pathophysiological roles of autophagy and aldo-keto reductases in development of doxorubicin resistance in gastrointestinal cancer cells. Chem. Biol. Interact..

[71] Jamrozik M., Piska K., Bucki A., Koczurkiewicz-Adamczyk P., Sapa M., Wladyka B., Pekala E., Kolaczkowski M. (2023). In Silico and In Vitro Assessment of Carbonyl Reductase 1 Inhibition Using ASP9521—A Potent Aldo-Keto Reductase 1C3 Inhibitor with the Potential to Support Anticancer Therapy Using Anthracycline Antibiotics. Molecules.

[72] Pinzon-Daza M.L., Cuellar-Saenz Y., Nualart F., Ondo-Mendez A., Del Riesgo L., Castillo-Rivera F., Garzon R. (2017). Oxidative Stress Promotes Doxorubicin-Induced Pgp and BCRP Expression in Colon Cancer Cells Under Hypoxic Conditions. J. Cell. Biochem..

[73] Jurj A., Ionescu C., Berindan-Neagoe I., Braicu C. (2022). The extracellular matrix alteration, implication in modulation of drug resistance mechanism: Friends or foes?. J. Exp. Clin. Cancer Res..

[74] Chen Z., Han F., Du Y., Shi H., Zhou W. (2023). Hypoxic microenvironment in cancer: Molecular mechanisms and therapeutic interventions. Signal Transduct. Target. Ther..

[75] Cheteh E.H., Sarne V., Ceder S., Bianchi J., Augsten M., Rundqvist H., Egevad L., Ostman A., Wiman K.G. (2020). Interleukin-6 derived from cancer-associated fibroblasts attenuates the p53 response to doxorubicin in prostate cancer cells. Cell Death Discov..

[76] Sun Y., Xiao L., Chen L., Wang X. (2025). Doxorubicin-Induced Cardiac Remodeling: Mechanisms and Mitigation Strategies. Cardiovasc. Drugs Ther..

[77] Pondugula S.R., Salamat J.M., Abbott K.L., Flannery P.C., Majrashi M., Almaghrabi M., Govindarajulu M., Ramesh S., Sandey M., Onteru S.K. (2021). A clinically relevant combination treatment with doxorubicin and cyclophosphamide does not induce hepatotoxicity in C57BL/6J mice. Liver Res..

[78] Amgalan D., Garner T.P., Pekson R., Jia X.F., Yanamandala M., Paulino V., Liang F.G., Corbalan J.J., Lee J., Chen Y. (2020). A small-molecule allosteric inhibitor of BAX protects against doxorubicin-induced cardiomyopathy. Nat. Cancer.

[79] Garner T.P., Amgalan D., Reyna D.E., Li S., Kitsis R.N., Gavathiotis E. (2019). Small-molecule allosteric inhibitors of BAX. Nat. Chem. Biol..

[80] Upshaw J.N., Parson S.K., Buchsbaum R.J., Schlam I., Ruddy K.J., Durani U., Epperla N., Leong D.P. (2024). Dexrazoxane to Prevent Cardiotoxicity in Adults Treated with Anthracyclines: JACC: CardioOncology Controversies in Cardio-Oncology. JACC CardioOncol..

[81] Hortobagyi G.N., Gutterman J.U., Blumenschein G.R., Tashima C.K., Burgess M.A., Einhorn L., Buzdar A.U., Richman S.P., Hersh E.M. (1979). Combination chemoimmunotherapy of metastatic breast cancer with 5-fluorouracil, adriamycin, cyclophosphamide, and BCG. Cancer.

[82] Nabholtz J., Mackey J., Smylie M., Paterson A., Noel D., Al-Tweigeri T., Tonkin K., North S., Azli N., Riva A. (2001). Phase II study of docetaxel, doxorubicin, and cyclophosphamide as first-line chemotherapy for metastatic breast cancer. J. Clin. Oncol..

[83] Santana-Krimskaya S.E., Franco-Molina M.A., Zarate-Trivino D.G., Prado-Garcia H., Zapata-Benavides P., Torres-Del-Muro F., Rodriguez-Padilla C. (2020). IMMUNEPOTENT CRP plus doxorubicin/cyclophosphamide chemotherapy remodel the tumor microenvironment in an air pouch triple-negative breast cancer murine model. Biomed. Pharmacother..

[84] Bukowski K., Kciuk M., Kontek R. (2020). Mechanisms of Multidrug Resistance in Cancer Chemotherapy. Int. J. Mol. Sci..

[85] Tuscano J.M., Martin S.M., Ma Y., Zamboni W., O’Donnell R.T. (2010). Efficacy, biodistribution, and pharmacokinetics of CD22-targeted pegylated liposomal doxorubicin in a B-cell non-Hodgkin’s lymphoma xenograft mouse model. Clin. Cancer Res..

[86] Wang J., Hu C., Wang J., Shen Y., Bao Q., He F., Wang H., Gong L., Liu Z., Hu F. (2019). Checkpoint Blockade in Combination with Doxorubicin Augments Tumor Cell Apoptosis in Osteosarcoma. J. Immunother..

[87] Merino M., Lozano T., Casares N., Lana H., Troconiz I.F., Ten Hagen T.L.M., Kochan G., Berraondo P., Zalba S., Garrido M.J. (2021). Dual activity of PD-L1 targeted Doxorubicin immunoliposomes promoted an enhanced efficacy of the antitumor immune response in melanoma murine model. J. Nanobiotechnol..

[88] Park H.J., Bae J.S., Kim K.M., Moon Y.J., Park S.-H., Ha S.H., Hussein U.K., Zhang Z., Park H.S., Park B.-H. (2018). The PARP inhibitor olaparib potentiates the effect of the DNA damaging agent doxorubicin in osteosarcoma. J. Exp. Clin. Cancer Res..

[89] Gehl J., Boesgaard M., Paaske T., Vittrup Jensen B., Dombernowsky P. (1996). Combined doxorubicin and paclitaxel in advanced breast cancer: Effective and cardiotoxic. Ann. Oncol..

[90] Gyongyosi M., Lukovic D., Zlabinger K., Spannbauer A., Gugerell A., Pavo N., Traxler D., Pils D., Maurer G., Jakab A. (2020). Liposomal doxorubicin attenuates cardiotoxicity via induction of interferon-related DNA damage resistance. Cardiovasc. Res..

[91] Fulton M.D., Najahi-Missaoui W. (2023). Liposomes in Cancer Therapy: How Did We Start and Where Are We Now. Int. J. Mol. Sci..

[92] Immordino M.L., Dosio F., Cattel L. (2006). Stealth liposomes: Review of the basic science, rationale, and clinical applications, existing and potential. Int. J. Nanomed..

[93] Livingston M.B., Jagosky M.H., Robinson M.M., Ahrens W.A., Benbow J.H., Farhangfar C.J., Foureau D.M., Maxwell D.M., Baldrige E.A., Begic X. (2021). Phase II Study of Pembrolizumab in Combination with Doxorubicin in Metastatic and Unresectable Soft-Tissue Sarcoma. Clin. Cancer Res..

[94] Lee E.K., Xiong N., Cheng S.C., Barry W.T., Penson R.T., Konstantinopoulos P.A., Hoffman M.A., Horowitz N., Dizon D.S., Stover E.H. (2020). Combined pembrolizumab and pegylated liposomal doxorubicin in platinum resistant ovarian cancer: A phase 2 clinical trial. Gynecol. Oncol..

[95] Nikolova M.P., Kumar E.M., Chavali M.S. (2022). Updates on Responsive Drug Delivery Based on Liposome Vehicles for Cancer Treatment. Pharmaceutics.

[96] Yi H., Lu W., Liu F., Zhang G., Xie F., Liu W., Wang L., Zhou W., Cheng Z. (2021). ROS-responsive liposomes with NIR light-triggered doxorubicin release for combinatorial therapy of breast cancer. J. Nanobiotechnol..

[97] Batist G., Ramakrishnan G., Rao C.S., Chandrasekharan A., Gutheil J., Guthrie T., Shah P., Khojasteh A., Nair M.K., Hoelzer K. (2001). Reduced cardiotoxicity and preserved antitumor efficacy of liposome-encapsulated doxorubicin and cyclophosphamide compared with conventional doxorubicin and cyclophosphamide in a randomized, multicenter trial of metastatic breast cancer. J. Clin. Oncol..

[98] Rafiyath S.M., Rasul M., Lee B., Wei G., Lamba G., Liu D. (2012). Comparison of safety and toxicity of liposomal doxorubicin vs. conventional anthracyclines: A meta-analysis. Exp. Hematol. Oncol..

[99] Ibrahim M., Abuwatfa W.H., Awad N.S., Sabouni R., Husseini G.A. (2022). Encapsulation, Release, and Cytotoxicity of Doxorubicin Loaded in Liposomes, Micelles, and Metal-Organic Frameworks: A Review. Pharmaceutics.

[100] Aloss K., Hamar P. (2023). Recent Preclinical and Clinical Progress in Liposomal Doxorubicin. Pharmaceutics.

[101] Sawant R.R., Torchilin V.P. (2012). Challenges in development of targeted liposomal therapeutics. AAPS J..

[102] Kumar Pramanik S., Losada-Pérez P., Reekmans G., Carleer R., D’Olieslaeger M., Vanderzande D., Adriaensens P., Ethirajan A. (2017). Physicochemical characterizations of functional hybrid liposomal nanocarriers formed using photo-sensitive lipids. Sci. Rep..

[103] Garcia-Pinel B., Porras-Alcala C., Ortega-Rodriguez A., Sarabia F., Prados J., Melguizo C., Lopez-Romero J.M. (2019). Lipid-Based Nanoparticles: Application and Recent Advances in Cancer Treatment. Nanomaterials.

[104] Fobian S.F., Cheng Z., Ten Hagen T.L.M. (2021). Smart Lipid-Based Nanosystems for Therapeutic Immune Induction against Cancers: Perspectives and Outlooks. Pharmaceutics.

[105] Patil Y.P., Jadhav S. (2014). Novel methods for liposome preparation. Chem. Phys. Lipids.

[106] Zhang H. (2017). Thin-Film Hydration Followed by Extrusion Method for Liposome Preparation. Liposomes.

[107] Nittayacharn P., Abenojar E., De Leon A., Wegierak D., Exner A.A. (2020). Increasing Doxorubicin Loading in Lipid-Shelled Perfluoropropane Nanobubbles via a Simple Deprotonation Strategy. Front. Pharmacol..

[108] Norouzi M., Yathindranath V., Thliveris J.A., Kopec B.M., Siahaan T.J., Miller D.W. (2020). Doxorubicin-loaded iron oxide nanoparticles for glioblastoma therapy: A combinational approach for enhanced delivery of nanoparticles. Sci. Rep..

[109] Kovrigina E., Chubarov A., Dmitrienko E. (2022). High Drug Capacity Doxorubicin-Loaded Iron Oxide Nanocomposites for Cancer Therapy. Magnetochemistry.

[110] Hoang Thi T.T., Nguyen Tran D.H., Bach L.G., Vu-Quang H., Nguyen D.C., Park K.D., Nguyen D.H. (2019). Functional Magnetic Core-Shell System-Based Iron Oxide Nanoparticle Coated with Biocompatible Copolymer for Anticancer Drug Delivery. Pharmaceutics.

[111] Goenka S., Sant V., Sant S. (2014). Graphene-based nanomaterials for drug delivery and tissue engineering. J. Control. Release.

[112] Liu J., Cui L., Losic D. (2013). Graphene and graphene oxide as new nanocarriers for drug delivery applications. Acta Biomater..

[113] Zainal-Abidin M.H., Hayyan M., Ngoh G.C., Wong W.F. (2020). Doxorubicin Loading on Functional Graphene as a Promising Nanocarrier Using Ternary Deep Eutectic Solvent Systems. ACS Omega.

[114] Mishra P., Dey R.K. (2018). Co-delivery of docetaxel and doxorubicin using biodegradable PEG-PLA micelles for treatment of breast cancer with synergistic anti-tumour effects. J. Macromol. Sci. Part A.

[115] Zhu Y., Zhang J., Meng F., Deng C., Cheng R., Feijen J., Zhong Z. (2016). cRGD-functionalized reduction-sensitive shell-sheddable biodegradable micelles mediate enhanced doxorubicin delivery to human glioma xenografts in vivo. J. Control. Release.

[116] Nittayacharn P., Nasongkla N. (2017). Development of self-forming doxorubicin-loaded polymeric depots as an injectable drug delivery system for liver cancer chemotherapy. J. Mater. Sci. Mater. Med..

[117] Sun J., Liu Y., Chen Y., Zhao W., Zhai Q., Rathod S., Huang Y., Tang S., Kwon Y.T., Fernandez C. (2017). Doxorubicin delivered by a redox-responsive dasatinib-containing polymeric prodrug carrier for combination therapy. J. Control. Release.

[118] Babanyinah G.K., Bhadran A., Polara H., Wang H., Shah T., Biewer M.C., Stefan M.C. (2024). Maleimide functionalized polycaprolactone micelles for glutathione quenching and doxorubicin delivery. Chem. Sci..

[119] Quiram G., Montagner F., Palmer K.L., Stefan M.C., Washington K.E., Rodrigues D.C. (2018). Novel Chlorhexidine-Loaded Polymeric Nanoparticles for Root Canal Treatment. J. Funct. Biomater..

[120] Bhadran A., Polara H., Calubaquib E.L., Wang H., Babanyinah G.K., Shah T., Anderson P.A., Saleh M., Biewer M.C., Stefan M.C. (2023). Reversible Cross-linked Thermoresponsive Polycaprolactone Micelles for Enhanced Stability and Controlled Release. Biomacromolecules.

[121] Washington K.E., Kularatne R.N., Du J., Gillings M.J., Webb J.C., Doan N.C., Biewer M.C., Stefan M.C. (2016). Synthesis of linear and star-like poly(ε-caprolactone)-b-poly{γ-2-[2-(2-methoxy-ethoxy)ethoxy]ethoxy-ε-caprolactone} amphiphilic block copolymers using zinc undecylenate. J. Polym. Sci. Part A Polym. Chem..

[122] Yang F., Xu J., Fu M., Ji J., Chi L., Zhai G. (2020). Development of stimuli-responsive intelligent polymer micelles for the delivery of doxorubicin. J. Drug Target..

[123] Liu Y., Ren Z., Zhang X., Zhao Z., Ma G., Pei Y., Zhao W., Wan D., Pan J. (2024). Dual-Triggered Peptide-Based Polymeric Micelles Enhance Doxorubicin Delivery for Targeted Cancer Therapy. ACS Appl. Nano Mater..

[124] Xu D.Z., Sun X.Y., Liang Y.X., Huang H.W., Liu R., Lu Z.L., He L. (2023). Esterase-Responsive Polymeric Micelles Containing Tetraphenylethene and Poly(ethylene glycol) Moieties for Efficient Doxorubicin Delivery and Tumor Therapy. Bioconjug. Chem..

[125] Veeren A., Bhaw-Luximon A., Mukhopadhyay D., Jhurry D. (2017). Mixed poly(vinyl pyrrolidone)-based drug-loaded nanomicelles shows enhanced efficacy against pancreatic cancer cell lines. Eur. J. Pharm. Sci..

[126] Sun Y., Zhang J., Han J., Tian B., Shi Y., Ding Y., Wang L., Han J. (2017). Galactose-Containing Polymer-DOX Conjugates for Targeting Drug Delivery. AAPS PharmSciTech.

[127] Chen K., Cai H., Zhang H., Zhu H., Gu Z., Gong Q., Luo K. (2019). Stimuli-responsive polymer-doxorubicin conjugate: Antitumor mechanism and potential as nano-prodrug. Acta Biomater..

[128] Xu L., Qiu L., Sheng Y., Sun Y., Deng L., Li X., Bradley M., Zhang R. (2018). Biodegradable pH-responsive hydrogels for controlled dual-drug release. J. Mater. Chem. B.

[129] Chittasupho C., Angklomklew J., Thongnopkoon T., Senavongse W., Jantrawut P., Ruksiriwanich W. (2021). Biopolymer Hydrogel Scaffolds Containing Doxorubicin as A Localized Drug Delivery System for Inhibiting Lung Cancer Cell Proliferation. Polymers.

[130] Chung C.K., Garcia-Couce J., Campos Y., Kralisch D., Bierau K., Chan A., Ossendorp F., Cruz L.J. (2020). Doxorubicin Loaded Poloxamer Thermosensitive Hydrogels: Chemical, Pharmacological and Biological Evaluation. Molecules.

[131] Wen Y., Li F., Li C., Yin Y., Li J. (2017). High mechanical strength chitosan-based hydrogels cross-linked with poly(ethylene glycol)/polycaprolactone micelles for the controlled release of drugs/growth factors. J. Mater. Chem. B.

[132] Chao Y., Liang Y., Fang G., He H., Yao Q., Xu H., Chen Y., Tang X. (2017). Biodegradable Polymersomes as Nanocarriers for Doxorubicin Hydrochloride: Enhanced Cytotoxicity in MCF-7/ADR Cells and Prolonged Blood Circulation. Pharm. Res..

[133] Albuquerque L.J.C., Sincari V., Jager A., Kucka J., Humajova J., Pankrac J., Paral P., Heizer T., Janouskova O., Davidovich I. (2021). pH-responsive polymersome-mediated delivery of doxorubicin into tumor sites enhances the therapeutic efficacy and reduces cardiotoxic effects. J. Control. Release.

[134] Goncalves M., Kairys V., Rodrigues J., Tomas H. (2022). Polyester Dendrimers Based on Bis-MPA for Doxorubicin Delivery. Biomacromolecules.

[135] Blanco E., Ferrari M. (2014). Emerging nanotherapeutic strategies in breast cancer. Breast.

[136] Valle J.W., Lawrance J., Brewer J., Clayton A., Corrie P., Alakhov V., Ranson M. (2004). A phase II, window study of SP1049C as first-line therapy in inoperable metastatic adenocarcinoma of the oesophagus. J. Clin. Oncol..

[137] Braccini S., Tacchini C., Chiellini F., Puppi D. (2022). Polymeric Hydrogels for In Vitro 3D Ovarian Cancer Modeling. Int. J. Mol. Sci..

[138] Parashar A.K., Saraogi G.K., Jain P.K., Kurmi B., Shrivastava V., Arora V. (2024). Polymer-drug conjugates: Revolutionizing nanotheranostic agents for diagnosis and therapy. Discov. Oncol..

[139] Kansız S., Elçin Y.M. (2023). Advanced liposome and polymersome-based drug delivery systems: Considerations for physicochemical properties, targeting strategies and stimuli-sensitive approaches. Adv. Colloid Interface Sci..

[140] Ekladious I., Colson Y.L., Grinstaff M.W. (2019). Polymer–drug conjugate therapeutics: Advances, insights and prospects. Nat. Rev. Drug Discov..

[141] Minehan R.L., Del Borgo M.P. (2022). Controlled release of therapeutics from enzyme-responsive biomaterials. Front. Biomater. Sci..

[142] Wang H., Polara H., Bhadran A., Shah T., Babanyinah G.K., Ma Z., Calubaquib E.L., Miller J.T., Biewer M.C., Stefan M.C. (2024). Effect of aromatic substituents on thermoresponsive functional polycaprolactone micellar carriers for doxorubicin delivery. Front. Pharmacol..

[143] Washington K.E., Kularatne R.N., Karmegam V., Biewer M.C., Stefan M.C. (2017). Recent advances in aliphatic polyesters for drug delivery applications. Wiley Interdiscip. Rev. Nanomed. Nanobiotechnol..

[144] Fentahun Darge H., Yibru Hanurry E., Simegniew Birhan Y., Worku Mekonnen T., Tizazu Andrgie A., Chou H.-Y., Lai J.-Y., Tsai H.-C. (2021). Multifunctional drug-loaded micelles encapsulated in thermo-sensitive hydrogel for in vivo local cancer treatment: Synergistic effects of anti-vascular and immuno-chemotherapy. Chem. Eng. J..

[145] Kim S., Shi Y., Kim J.Y., Park K., Cheng J.X. (2010). Overcoming the barriers in micellar drug delivery: Loading efficiency, in vivo stability, and micelle-cell interaction. Expert Opin. Drug Deliv..

[146] Cai K., He X., Song Z., Yin Q., Zhang Y., Uckun F.M., Jiang C., Cheng J. (2015). Dimeric drug polymeric nanoparticles with exceptionally high drug loading and quantitative loading efficiency. J. Am. Chem. Soc..

[147] Liao C., Chen Y., Yao Y., Zhang S., Gu Z., Yu X. (2016). Cross-Linked Small-Molecule Micelle-Based Drug Delivery System: Concept, Synthesis, and Biological Evaluation. Chem. Mater..

[148] Della Rocca J., Liu D., Lin W. (2012). Are high drug loading nanoparticles the next step forward for chemotherapy?. Nanomedicine.

[149] Ahmad Z., Shah A., Siddiq M., Kraatz H.-B. (2014). Polymeric micelles as drug delivery vehicles. RSC Adv..

[150] Yang C., Attia A.B., Tan J.P., Ke X., Gao S., Hedrick J.L., Yang Y.Y. (2012). The role of non-covalent interactions in anticancer drug loading and kinetic stability of polymeric micelles. Biomaterials.

[151] McLaughlin C.K., Logie J., Shoichet M.S. (2013). Core and Corona Modifications for the Design of Polymeric Micelle Drug-Delivery Systems. Isr. J. Chem..

[152] Zhuang W.R., Wang Y., Cui P.F., Xing L., Lee J., Kim D., Jiang H.L., Oh Y.K. (2019). Applications of π-π stacking interactions in the design of drug-delivery systems. J. Control. Release.

[153] Li Y., Su T., Li S., Lai Y., He B., Gu Z. (2014). Polymeric micelles with π-π conjugated moiety on glycerol dendrimer as lipophilic segments for anticancer drug delivery. Biomater. Sci..

[154] Zhang H., Yan J., Mei H., Cai S., Li S., Cheng F., Cao J., He B. (2020). High-drug-loading capacity of redox-activated biodegradable nanoplatform for active targeted delivery of chemotherapeutic drugs. Regen. Biomater..

[155] Li Z., Liu J., Sun Z., Li Y., Yu B., Zhao F., Wang H., Xu H. (2022). Nanomicelles co-loaded with doxorubicin and salvianolic acid A for breast cancer chemotherapy. Cancer Nanotechnol..

[156] Bhadran A., Shah T., Babanyinah G.K., Polara H., Taslimy S., Biewer M.C., Stefan M.C. (2023). Recent Advances in Polycaprolactones for Anticancer Drug Delivery. Pharmaceutics.

[157] Wang H., Calubaquib E.L., Bhadran A., Ma Z., Miller J.T., Zhang A., Biewer M.C., Stefan M.C. (2023). Self-assembly behavior of thermoresponsive difunctionalized γ-amide polycaprolactone amphiphilic diblock copolymers. Polym. Chem..

[158] Hao J., Cheng Y., Ranatunga R.J.K.U., Senevirathne S., Biewer M.C., Nielsen S.O., Wang Q., Stefan M.C. (2013). A Combined Experimental and Computational Study of the Substituent Effect on Micellar Behavior of γ-Substituted Thermoresponsive Amphiphilic Poly(ε-caprolactone)s. Macromolecules.

[159] Shah T., Polara H., Babanyinah G., Bhadran A., Wang H., Castillo C.C., Grabowski G., Biewer M.C., Torabifard H., Stefan M.C. (2025). Computational design to experimental validation: Molecular dynamics-assisted development of polycaprolactone micelles for drug delivery. J. Mater. Chem. B.

[160] Babanyinah G.K., Bhadran A., Polara H., Shah T., Biewer M.C., Stefan M.C. (2025). Fluorescent Poly(ε-Caprolactone)s Micelles for Anticancer Drug Delivery and Bioimaging. Biomacromolecules.

[161] Polara H., Shah T., Babanyinah G., Wang H., Bhadran A., Biewer M.C., Stefan M.C. (2025). Improved Drug Delivery through Amide-Functionalized Polycaprolactones: Enhanced Loading Capacity and Sustained Drug Release. Biomacromolecules.

[162] Washington K.E., Kularatne R.N., Biewer M.C., Stefan M.C. (2018). Combination Loading of Doxorubicin and Resveratrol in Polymeric Micelles for Increased Loading Efficiency and Efficacy. ACS Biomater. Sci. Eng..

[163] Soltantabar P., Calubaquib E.L., Mostafavi E., Biewer M.C., Stefan M.C. (2020). Enhancement of loading efficiency by coloading of doxorubicin and quercetin in thermoresponsive polymeric micelles. Biomacromolecules.

[164] Zeng W., Luo Y., Gan D., Zhang Y., Deng H., Liu G. (2023). Advances in Doxorubicin-based nano-drug delivery system in triple negative breast cancer. Front. Bioeng. Biotechnol..

[165] Sun W., Fan J., Wang S., Kang Y., Du J., Peng X. (2018). Biodegradable Drug-Loaded Hydroxyapatite Nanotherapeutic Agent for Targeted Drug Release in Tumors. ACS Appl. Mater. Interfaces.

[166] Jaimes-Aguirre L., Morales-Avila E., Ocampo-Garcia B.E., Medina L.A., Lopez-Tellez G., Gibbens-Bandala B.V., Izquierdo-Sanchez V. (2017). Biodegradable poly(D,L-lactide-co-glycolide)/poly(L-γ-glutamic acid) nanoparticles conjugated to folic acid for targeted delivery of doxorubicin. Mater. Sci. Eng. C Mater. Biol. Appl..

[167] Senevirathne S.A., Washington K.E., Miller J.B., Biewer M.C., Oupicky D., Siegwart D.J., Stefan M.C. (2017). HDAC Inhibitor Conjugated Polymeric Prodrug Micelles for Doxorubicin Delivery. J. Mater. Chem. B.

[168] Calubaquib E.L., Soltantabar P., Wang H., Shin H., Flores A., Biewer M.C., Stefan M.C. (2021). Self-assembly behavior of oligo(ethylene glycol) substituted polycaprolactone homopolymers. Polym. Chem..

[169] Hashem S., Ali T.A., Akhtar S., Nisar S., Sageena G., Ali S., Al-Mannai S., Therachiyil L., Mir R., Elfaki I. (2022). Targeting cancer signaling pathways by natural products: Exploring promising anti-cancer agents. Biomed. Pharmacother..

[170] Nasr M., Hashem F., Teiama M., Tantawy N., Abdelmoniem R. (2024). Folic acid grafted mixed polymeric micelles as a targeted delivery strategy for tamoxifen citrate in treatment of breast cancer. Drug Deliv. Transl. Res..

[171] An H., Yang Y., Zhou Z., Bo Y., Wang Y., He Y., Wang D., Qin J. (2021). Pectin-based injectable and biodegradable self-healing hydrogels for enhanced synergistic anticancer therapy. Acta Biomater.

[172] Cao J., Du X., Zhao H., Zhu C., Li C., Zhang X., Wei L., Ke X. (2023). Sequentially degradable hydrogel-microsphere loaded with doxorubicin and pioglitazone synergistically inhibits cancer stemness of osteosarcoma. Biomed. Pharmacother..

[173] Alipournazari P., Pourmadadi M., Abdouss M., Rahdar A., Pandey S. (2024). Enhanced delivery of doxorubicin for breast cancer treatment using pH-sensitive starch/PVA/g-C3N4 hydrogel. Int. J. Biol. Macromol..

[174] Akkaya B., Akkaya R., Celikkaya S.I., Sarıaydin N., Raheem K.Y. (2023). Doxorubucin loaded pH-responsive chitosan-poly(acrylamide-maleic acid) composite hydrogel for anticancer targeting. J. Mol. Struct..

[175] Nikzamir M., Hanifehpour Y., Akbarzadeh A., Panahi Y. (2021). Applications of dendrimers in nanomedicine and drug delivery: A review. J. Inorg. Organomet. Polym. Mater..

[176] Kaurav M., Ruhi S., Al-Goshae H.A., Jeppu A.K., Ramachandran D., Sahu R.K., Sarkar A.K., Khan J., Ashif Ikbal A.M. (2023). Dendrimer: An update on recent developments and future opportunities for the brain tumors diagnosis and treatment. Front. Pharmacol..

[177] Soltany P., Miralinaghi M., Shariati F.P. (2024). Folic acid conjugated poly (Amidoamine) dendrimer grafted magnetic chitosan as a smart drug delivery platform for doxorubicin: In-vitro drug release and cytotoxicity studies. Int. J. Biol. Macromol..

[178] Szota M., Jachimska B. (2023). Effect of Alkaline Conditions on Forming an Effective G4. 0 PAMAM Complex with Doxorubicin. Pharmaceutics.

[179] Zhang H., Cui W., Qu X., Wu H., Qu L., Zhang X., Mäkilä E., Salonen J., Zhu Y., Yang Z. (2019). Photothermal-responsive nanosized hybrid polymersome as versatile therapeutics codelivery nanovehicle for effective tumor suppression. Proc. Natl. Acad. Sci. USA.

[180] Ferrero C., Casas M., Caraballo I. (2022). Redox-Responsive Polymersomes as Smart Doxorubicin Delivery Systems. Pharmaceutics.

[181] Nehate C., Nayal A., Koul V. (2018). Redox responsive polymersomes for enhanced doxorubicin delivery. ACS Biomater. Sci. Eng..

[182] Bobde Y., Patel T., Paul M., Biswas S., Ghosh B. (2021). PEGylated N-(2 hydroxypropyl) methacrylamide polymeric micelles as nanocarriers for the delivery of doxorubicin in breast cancer. Colloids Surf. B: Biointerfaces.

[183] Janani S., John J., Viswanad V. (2025). Poly (N-(2-hydroxypropyl) methacrylamide). Synthetic Polymers in Drug and Biotherapeutics Delivery.

[184] Braunová A., Chytil P., Laga R., Šírová M., Machová D., Parnica J., Říhová B., Janoušková O., Etrych T. (2020). Polymer nanomedicines based on micelle-forming amphiphilic or water-soluble polymer-doxorubicin conjugates: Comparative study of in vitro and in vivo properties related to the polymer carrier structure, composition, and hydrodynamic properties. J. Control. Release.

[185] Ou Y., Chen K., Cai H., Zhang H., Gong Q., Wang J., Chen W., Luo K. (2018). Enzyme/pH-sensitive polyHPMA–DOX conjugate as a biocompatible and efficient anticancer agent. Biomater. Sci..

[186] Bajwa N., Mahal S., Singh P.A., Jyoti K., Dewangan P., Madan J., Baldi A. (2023). Drug–polymer conjugates: Challenges, opportunities, and future prospects in clinical trials. Polym. Drug Conjug..

[187] Parshad B., Arora S., Singh B., Pan Y., Tang J., Hu Z., Patra H.K. (2025). Towards precision medicine using biochemically triggered cleavable conjugation. Commun. Chem..

[188] Singh D. (2024). Dynamic Covalent Macromolecular Networks for Adaptive Drug Delivery Systems: An Informative Review. J. Macromol. Sci. Part B.

[189] Utterström J., Naeimipour S., Selegård R., Aili D. (2021). Coiled coil-based therapeutics and drug delivery systems. Adv. Drug Deliv. Rev..

[190] Kwon G.S. (2003). Polymeric micelles for delivery of poorly water-soluble compounds. Crit. Rev. Ther. Drug Carr. Syst..

[191] Shi Y., Lammers T., Storm G., Hennink W.E. (2017). Physico-Chemical Strategies to Enhance Stability and Drug Retention of Polymeric Micelles for Tumor-Targeted Drug Delivery. Macromol. Biosci..

[192] D’Addio S.M., Saad W., Ansell S.M., Squiers J.J., Adamson D.H., Herrera-Alonso M., Wohl A.R., Hoye T.R., Macosko C.W., Mayer L.D. (2012). Effects of block copolymer properties on nanocarrier protection from in vivo clearance. J. Control. Release.

[193] Lila A.S.A., Kiwada H., Ishida T. (2013). The accelerated blood clearance (ABC) phenomenon: Clinical challenge and approaches to manage. J. Control. Release.

[194] Walkey C.D., Chan W.C.W. (2012). Understanding and controlling the interaction of nanomaterials with proteins in a physiological environment. Chem. Soc. Rev..

[195] Cedervall T., Lynch I., Lindman S., Berggård T., Thulin E., Nilsson H., Dawson K.A., Linse S. (2007). Understanding the nanoparticle–protein corona using methods to quantify exchange rates and affinities of proteins for nanoparticles. Proc. Natl. Acad. Sci. USA.

